# Association of oxidative balance score with cardiovascular disease and all-cause and cardiovascular mortality in American adults with type 2 diabetes: data from the National Health and Nutrition examination survey 1999-2018

**DOI:** 10.3389/fendo.2024.1458039

**Published:** 2024-12-16

**Authors:** Meilin Fan, Shina Song, Tingting Chu, Ronghong Li, Miao Yue, Xiaofeng Li, Jing Yang

**Affiliations:** ^1^ Department of Endocrinology, First Hospital of Shanxi Medical University, Taiyuan, China; ^2^ Department of Endocrinology, Linfen City People’s Hospital, Linfen, China; ^3^ Department of Geriatrics, General Hospital of TISCO, Taiyuan, China; ^4^ Department of Neurology, First Hospital of Shanxi Medical University, Taiyuan, China; ^5^ Department of Cardiology, Linfen City People’s Hospital, Linfen, China; ^6^ Department of General Medicine, Linfen City People’s Hospital, Linfen, China

**Keywords:** oxidative balance score, type 2 diabetes, oxidative stress, mortality, cardiovascular disease

## Abstract

**Background:**

Oxidative stress has an important role in type 2 diabetes (T2D). Oxidative balance score (OBS) is an emerging assessment of dietary and lifestyle oxidative balance. We aimed to explore the association of OBS with cardiovascular disease (CVD) and all-cause and CVD mortality in the T2D population through NHANES 1999-2018.

**Methods:**

OBS integrated 16 dietary components and 4 lifestyle components. T2D was diagnosed according to the American Diabetes Association criteria. Multivariate logistic regression and multivariate Cox proportional hazards regression analyses were used to explore the association of OBS with CVD and mortality in T2D, respectively.

**Results:**

3801 adult T2D participants were included. In fully adjusted models, OBS, dietary OBS, and lifestyle OBS were all negatively associated with the prevalence of CVD (odds ratios of 0.98, 0.98, and 0.85, respectively). Higher OBS and lifestyle OBS (p for trend 0.016 and <0.001, respectively) rather than dietary OBS (p for trend = 0.06) were associated with significantly lower odds of CVD. Higher OBS, dietary OBS, and lifestyle OBS were all negatively associated with all-cause mortality (hazard ratios [HR] of 0.98, 0.98, and 0.92, respectively; p for trend of 0.002, 0.009, and 0.035, respectively). Higher OBS and dietary OBS were negatively associated with CVD mortality (HR 0.96 and 0.95, respectively; p for trend both <0.001), whereas lifestyle OBS was not. Restricted cubic spline analysis suggested that most associations were linear. Stratified analyses showed that these associations were influenced by some demographic variables and disease status.

**Conclusions:**

Adherence to higher OBS was associated with reduced CVD prevalence and mortality risk in T2D. Antioxidant diet and lifestyle had more significant associations with mortality and CVD prevalence, respectively. However, as these findings are merely associations and do not allow causal inferences to be drawn, future validation in high-quality randomized controlled trials is needed.

## 1 Introduction

Diabetes is one of the most common chronic non-communicable diseases worldwide and is recognized as one of the global public health challenges. The International Diabetes Federation Diabetes Atlas estimated that approximately 536.6 million adult people worldwide had diabetes in 2021 and is projected to increase to 783.2 million people in 2045 (1). Diabetes and its complications and mortality are associated with diminished quality of life and a heavy economic burden, with global diabetes-related healthcare expenditures in 2021 estimated at $966 billion (1, 2). Type 2 diabetes (T2D) accounts for most cases of diabetes and is highly prevalent in middle-aged and older adults (3). T2D is a well-recognized risk factor for incident cardiovascular disease (CVD) and is associated with increased mortality. CVD is the leading cause of death in the T2D population and poses a heavy economic burden (4, 5). About one-third of the T2D population is affected by CVD, and about half of all deaths come from CVD (6). CVD incidence and mortality in the T2D population have generally shown a decreasing trend over the past decades, but there is an increasing trend in low-middle-income countries (7, 8). Given that the burden of disease remains significantly higher in T2D than in non-T2D populations (7), appropriate interventions need to be explored to improve prognosis in T2D populations.

Although the mechanisms underlying the onset and progression of T2D are still not fully understood, cumulative experimental studies suggest that oxidative stress is an important hallmark of T2D pathogenesis. Hyperglycemia-induced oxidative stress is associated with impairment of insulin signaling pathways and may have an important role in diabetes-related complications (9). Oxidative stress may serve as an important molecular mechanism to promote CVD and poor clinical prognosis in T2D (10). Dietary or lifestyle sources of antioxidants and pro-oxidants have been suggested to have the potential to modulate intrinsic oxidative balance. It is well recognized that there is little open question about the association between a healthy diet/lifestyle and a reduced prevalence of CVD in the general population, although inferences of causality are still lacking. However, current evidence on the association of dietary and lifestyle sources of antioxidants and pro-oxidants with CVD and prognosis in T2D populations remains limited. Most previous studies have focused on the association of certain individual dietary or lifestyle anti/pro-oxidants with mortality in T2D populations, and inconsistent conclusions were noted (11–14). A recent study from the National Health and Nutrition Examination Survey (NHANES) suggested that integrated dietary antioxidant intake was associated with a reduced risk of mortality in the T2D population, while another study using NHANES did not yield similar findings (11, 12). Physical activity as a lifestyle antioxidant has been shown to be associated with a reduced risk of mortality in T2D populations, whereas smoking as a pro-oxidant has been associated with an increased risk of mortality in diabetic populations (13, 14). Given the controversial nature of the previous findings, there is an urgent need for research addressing the association of integrated dietary and lifestyle antioxidant and pro-oxidant exposures with CVD and mortality in patients with T2D.

The oxidative balance score (OBS) is an emerging composite oxidative balance assessment metric that integrates dietary and lifestyle sources of antioxidants and pro-oxidants (15). Compared to individual antioxidants/pro-oxidants, OBS comprehensively accounts for the combined effects of dietary and lifestyle pro-oxidants and antioxidants and more accurately reflects an individual’s exposure to oxidative stress. A large body of clinical evidence has demonstrated that OBS is associated with the development and or prognosis of a range of diseases (15). Several previous observational clinical studies from NHANES have suggested that OBS as an emerging integrated assessment of diet and lifestyle may be associated with reduced odds of CVD and lower 10-year atherosclerotic CVD risk in the general population (16–18). However, the association of OBS with CVD risk in specific populations is controversial. One study from NHANES showed that OBS was associated with reduced odds of CVD in patients with nonalcoholic fatty liver disease (NAFLD) (19), while another cohort study did not demonstrate an association between OBS and CVD risk in a population with chronic renal insufficiency (20). In addition, cumulative observational evidence suggests that higher OBS is associated with a reduced risk of mortality in the general population or in specific (e.g., NAFLD, metabolic syndrome) populations (19, 21, 22). Of note, a previous study showed that OBS was associated with the odds of T2D in the general population (23). However, there is a dearth of real-world studies demonstrating the association of OBS with CVD prevalence and mortality in the T2D population.

In this study, we aimed to explore the association of OBS with CVD prevalence and mortality in people with T2D using nationally representative data from NHANES. Our findings reveal that OBS may have an important role in CVD and mortality prevention in T2D populations. Our study suggests that OBS may be of public health importance as a modifiable risk factor in the mitigation of CVD and mortality disease burden in T2D.

## 2 Methods

### 2.1 Study design and population

NHANES is the primary program of the National Center for Health Statistics (NCHS) dedicated to assessing the health and nutritional status of noninstitutionalized populations and providing vital epidemiologic statistics. Since 1999, NHANES has been a continuous program with a two-year cycle. NHANES consists of a series of publicly accessible questionnaires and physical examination data. NHANES is a serial, nationally representative, complex, cross-sectional survey with a multistage probability sampling design. All NHANES survey protocols were approved by the NCHS Ethics Review Board, and all participants provided written informed consent.

**Figure 1:**
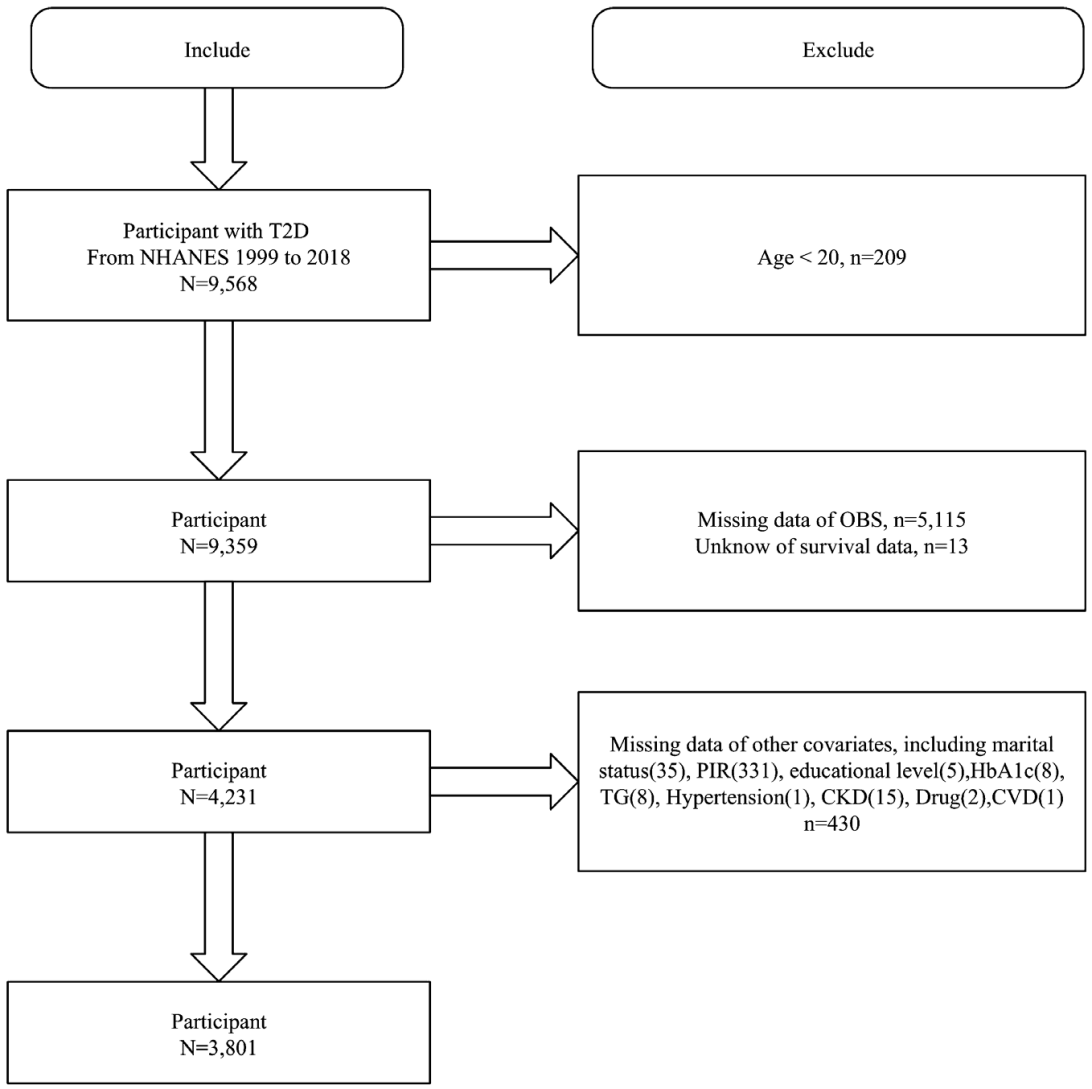
Flowchart of study population selection, NHANES 1999-2018.

We first included 9,568 T2D participants from NHANES 1999-2018 and excluded those aged <20 years (n=209), missing OBS (n=5115), unknown survival data (n=13), and missing covariates (n=430). Finally, 3801 T2D participants were included (**Figure 1**).

### 2.2 OBS components and assessment

The specific OBS components and assignment modalities were presented in **Supplementary Table S1**. In this study, we assessed the OBS using component and assignment criteria that have been extensively validated in previous studies (24–26). The use of OBS in NHANES was first proposed by Zhang et al. which expanded the previous OBS components into a dietary OBS containing 16 components and a lifestyle OBS with 4 components (27). These components were added based on the antioxidant properties of the corresponding components demonstrated in the literature (27). According to previous NHANES-related studies, no specific weights were assigned to the components of the OBS, i.e., each component was uniformly assigned a score based on level (26, 27). Most of these components were assigned scores based on the gender-specific tertile of their values (antioxidants: 0 for the lowest tertile T1, 1 for T2, and 2 for the highest tertile; the opposite was true for pro-oxidants). The alcohol intake component was categorized according to widely accepted criteria as non-drinkers (2 points), non-heavy drinkers (1 point), and heavy drinkers (0 points). Thus, the respective cutoff values in the OBS assignments were actually determined by the sex-specific tertiles of the respective components. The OBS consisted of 16 dietary components (14 antioxidants, including dietary fiber, carotenoids, riboflavin, niacin, vitamin B6, total folate, vitamin B12, vitamin C, vitamin E, calcium, magnesium, zinc, copper, and selenium, and 2 pro-oxidants, including total fat and iron) and 4 lifestyle components (1 antioxidant, namely physical activity, and 3 pro-oxidants, including body mass index [BMI], alcohol consumption, and serum cotinine exposure). Dietary intake information was obtained from the average of two 24-hour dietary recall questionnaires, and dietary nutrient intake was obtained from the USDA Food and Nutrient Database for Dietary Studies (25). Physical activity was assessed according to metabolic equivalents [MET], the final amount of physical activity was the number of minutes of activity per week multiplied by the MET score for each activity (28). BMI was derived by dividing weight (in kilograms) by the square of height (in meters), as determined by skilled staff at the Mobile Examination Center. Serum cotinine was considered as a proxy for active and passive smoking exposure. Alcohol intake (g/d) was self-reported from the relevant dietary recall questionnaire (ALQ). Overall, antioxidants and pro-oxidants were assigned a score based on sex-specific tertile taking (0-2 points). Alcohol intake was scored according to the criteria of previous studies (men: 0, 1, and 2 points for >30/0-30/0 g/d, respectively; women: 0, 1, and 2 points for >15/0-1/0 g/d, respectively) (24).

### 2.3 T2D diagnosis

We assessed T2D according to the American Diabetes Association (ADA) criteria (29). The presence of diabetes was indicated by one of the following: self-reported history of diabetes, fasting blood glucose ≥7.0 mmol/L, 2-h oral glucose tolerance test blood glucose or random blood glucose ≥11.1 mmol/L, glycated hemoglobin A1c (HbA1c) ≥6.5%, or taking antidiabetic medications (29). This criterion has been validated in numerous previous high-quality studies using NHANES (30, 31).

### 2.4 CVD assessment

CVD (coronary heart disease, congestive heart failure, angina, stroke, or heart attack) was diagnosed based on self-reporting in the questionnaire (32). Participants were asked: “Has a doctor or other health professional ever told you that you had a coronary heart disease/congestive heart failure/angina/stroke/heart attack?”, and affirmative responses indicated the presence of that type of CVD.

### 2.5 Mortality data collection

We prospectively followed the T2D population at baseline through December 31, 2019, to obtain mortality outcomes. Mortality data were obtained through public-use linked mortality files from the National Death Index database. CVD mortality was accessed through ICD-10 codes related to cardiac and cerebrovascular disease deaths, including I00-I09, I11, I13, I20-I51, and I60-I69.

### 2.6 Covariates

We included age, gender, race/ethnicity, educational attainment, household income-poverty ratio (PIR), marital status, HbA1c, antidiabetic medication use, hypertension, CVD (adjusted when exploring OBS and mortality in T2D), chronic kidney disease (CKD), serum triglycerides (TG), total cholesterol (TC), and high-density lipoprotein cholesterol (HDL-C) according to prior relevant studies (11, 12, 33, 34). Antidiabetic medication use or not was obtained based on self-report of relevant questionnaires (11). Hypertension was diagnosed based on a self-reported history of hypertension, a blood pressure value of ≥140/90 mmHg, or the use of antihypertensive medications (35). According to the KDIGO 2021 clinical practice guideline, CKD was defined as having a urinary albumin/creatinine ratio ≥ 30 mg/g and/or an estimated glomerular filtration rate (eGFR) < 60 ml/min/1.73 m^2^, where eGFR was calculated according to the Chronic Kidney Disease Epidemiology Collaborative group equation (36, 37). Serum lipid profiles were obtained from biochemistry profiles of laboratory tests in NHANES.

### 2.7 Statistical analysis

We weighted all analyses according to NHANES analysis guidelines where relevant to account for the complex study design of NHANES. Statistical analyses were performed using R (version 4.2.3), and a two-sided p-value of less than 0.05 indicated statistical significance. Baseline analysis was performed by grouping the T2D population according to OBS quartiles. Continuous variables were expressed as mean ± standard error and tested for between-group differences by ANOVA; categorical variables were expressed as number (percentage) and tested by chi-square analysis. The association between OBS and CVD prevalence in the T2D population was analyzed using multivariate logistic regression models. We constructed multiple adjusted models, where the crude model did not adjust for any covariates; model 1 adjusted for age, sex, race/ethnicity, education, PIR, and marital status; and model 2 additionally adjusted for HbA1c, antidiabetic medication use, hypertension, CKD, TG, TC, and HDL-C from model 1. Kaplan-Meier (KM) survival analyses were used to explore differences in all-cause and CVD-related survival probabilities in the T2D population across OBS quartiles. Multivariate Cox proportional hazards regression models were used to explore the association between OBS and mortality in the T2D population. Similarly, the crude model did not adjust for any covariates; model 1 adjusted for age, sex, race/ethnicity, PIR, education, and marital status; and model 2 additionally adjusted for HbA1c, antidiabetic medication use, CVD, hypertension, CKD, TG, TC, and HDL-C based on model 1. Restricted cubic spline (RCS) modeling was used to explore potential nonlinear associations or dose-response associations and select appropriate knots for smooth curve fitting. Stratified analyses were conducted to reveal whether these associations remained constant across subgroups and interaction analyses were conducted to identify effect modifiers. In sensitivity analyses, we additionally adjusted for diabetes duration to verify the stability of the findings.

## 3 Results

### 3.1 Baseline characteristics

3801 T2D participants were included with a mean age of 57.40 years and a mean OBS score of 20.30. T2D individuals with higher OBS had higher PIR and HDL-C, lower TG and TC, and were more likely to be of non-Hispanic White race/ethnicity, non-single, and greater than a high school education. In addition, as OBS increased, the prevalence of CVD and CKD was lower in the T2D population (**Table 1**).

**Table 1:** Baseline analysis of the T2D population according to OBS quartiles.

	Total (n=3801)	Q1 (n=1064)	Q2 (n=848)	Q3 (n=1027)	Q4 (n=862)	P-value
**OBS**	20.3 ± 0.2	11.0 ± 0.1	17.2 ± 0.1	22.4 ± 0.1	28.7 ± 0.1	<0.0001
**OBS dietary**	16.4 ± 0.2	7.6 ± 0.1	13.3 ± 0.1	18.4 ± 0.1	24.2 ± 0.1	<0.0001
**OBS lifestyle**	3.9 ± 0.0	3.3 ± 0.0	3.9 ± 0.1	4.0 ± 0.1	4.4 ± 0.1	<0.0001
**Age, year**	57.4 ± 0.3	57.5 ± 0.6	57.2 ± 0.6	57.3 ± 0.5	57.5 ± 0.6	0.97
**PIR**	3.0 ± 0.0	2.5 ± 0.1	2.8 ± 0.1	3.2 ± 0.1	3.4 ± 0.1	<0.0001
**HbA1c, %**	7.0 ± 0.0	7.1 ± 0.1	7.0 ± 0.1	7.0 ± 0.1	7.0 ± 0.1	0.35
**TG, mg/dL**	197 ± 4	203 ± 8	182 ± 7	211 ± 9	191 ± 8	0.03
**TC, mg/dL**	189 ± 1	193 ± 3	191 ± 2	190 ± 2	183 ± 2	0.002
**HDL-C, mg/dL**	48 ± 0	48 ± 1	48 ± 1	46 ± 1	49 ± 1	0.002
**Sex**						0.82
male	2113 (54.6)	641 (56.3)	476 (55.4)	546 (53.4)	450 (53.9)	
female	1688 (45.4)	423 (43.7)	372 (44.6)	481 (46.6)	412 (46.1)	
**Race/ethnicity**						<0.0001
Mexican American	746 (8.6)	195 (8.0)	177 (9.1)	192 (8.6)	182 (8.6)	
Non-Hispanic Black	868 (12.6)	325 (19.8)	199 (13.6)	196 (10.3)	148 (8.2)	
Non-Hispanic White	1529 (66.2)	393 (60.4)	326 (65.8)	438 (66.6)	372 (71.0)	
Other Hispanic	329 (5.5)	85 (6.1)	79 (5.3)	91 (6.0)	74 (4.7)	
Other Race	329 (7.1)	66 (5.7)	67 (6.2)	110 (8.5)	86 (7.5)	
**Marital Status**						0.01
non-single	2426 (67.2)	644 (61.2)	534 (67.8)	668 (67.2)	580 (72.1)	
single	1375 (32.8)	420 (38.8)	314 (32.2)	359 (32.8)	282 (27.9)	
**Education**						<0.0001
<high school	517 (6.7)	195 (10.3)	141 (8.1)	115 (5.9)	66 (3.2)	
high school	1491 (37.2)	485 (46.8)	333 (38.8)	363 (32.6)	310 (32.6)	
>high school	1793 (56.1)	384 (42.9)	374 (53.1)	549 (61.5)	486 (64.2)	
**Hypertension**						0.43
no	1183 (33.2)	289 (30.1)	267 (32.7)	345 (34.8)	282 (34.4)	
yes	2618 (66.8)	775 (69.9)	581 (67.3)	682 (65.2)	580 (65.6)	
**CVD**						0.01
no	2988 (80.7)	773 (75.9)	682 (81.0)	818 (82.6)	715 (82.6)	
yes	813 (19.3)	291 (24.1)	166 (19.0)	209 (17.4)	147 (17.4)	
**CKD**						<0.001
no	2466 (68.8)	647 (62.5)	535 (65.4)	694 (71.1)	590 (74.6)	
yes	1335 (31.2)	417 (37.5)	313 (34.6)	333 (28.9)	272 (25.4)	
**Anti-Diabetic drugs**						0.24
no	1506 (39.8)	445 (43.7)	345 (39.4)	365 (37.3)	351 (39.4)	
yes	2295 (60.2)	619 (56.3)	503 (60.6)	662 (62.7)	511 (60.6)	

Continuous variables were expressed as mean ± standard error and tested for between-group differences by ANOVA; categorical variables were expressed as number (percentage) and tested by chi-square analysis. T2D, type 2 diabetes; OBS, oxidative balance score; PIR, income-poverty ratio; HbA1c, glycated hemoglobin A1c; TG, triglycerides; TC, total cholesterol; HDL-C, high-density lipoprotein cholesterol; CVD, cardiovascular disease; CKD, chronic kidney disease.

### 3.2 Association of OBS with CVD prevalence in the T2D population

**Figure 2:**
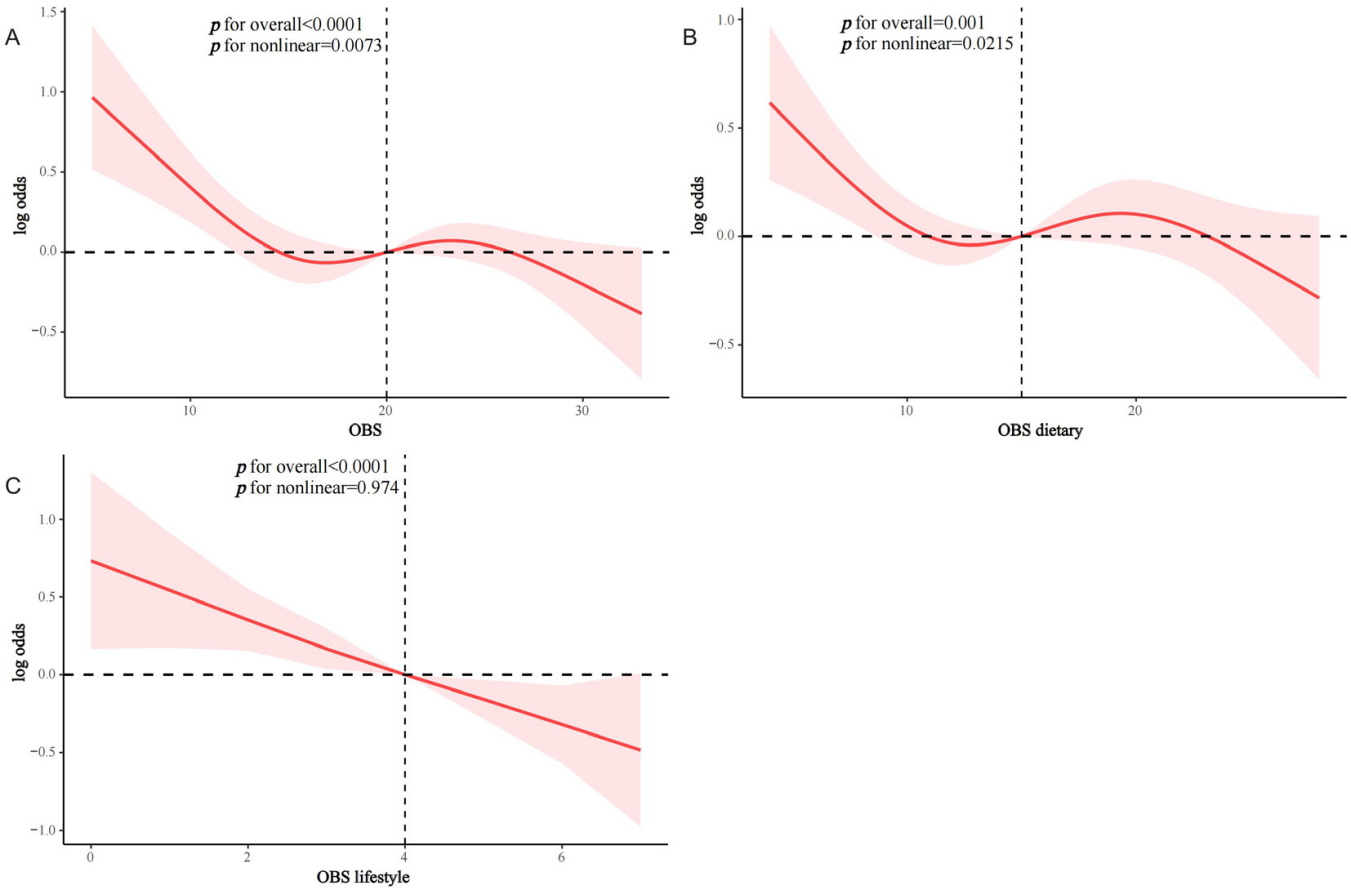
RCS analysis of OBS and the prevalence of CVD in the T2D population. **(A)** OBS; **(B)** dietary OBS; **(C)** lifestyle OBS.

OBS, dietary OBS, and lifestyle OBS were all negatively associated with CVD prevalence in T2D in both the crude model and Model 1. After adjusting for all confounders, OBS, dietary OBS, and lifestyle OBS remained inversely associated with the odds of CVD (OBS: odds ratio [OR] and 95% confidence interval [CI] = 0.98 (0.96,0.99), p = 0.006; dietary OBS: OR and 95% CI = 0.98 (0.96,1.00), p = 0.032; lifestyle OBS: OR and 95% CI = 0.85 (0.78,0.91), p < 0.0001). Higher OBS and lifestyle OBS were associated with significantly lower CVD prevalence (p for trend 0.016 and <0.001, respectively), while a similar trend was present with dietary OBS (p for trend = 0.06) (**Table 2**). RCS modeling indicated that OBS and dietary OBS were nonlinearly associated with the prevalence of CVD (p for nonlinearity = 0.0073 and 0.0215), whereas lifestyle OBS was linearly associated with the odds of CVD (p for nonlinearity = 0.974) (**Figure 2**).

**Table 2:** Association of OBS with CVD prevalence in the T2D population.

	Crude Model OR (95%CI)	P-value	Model 1 OR (95%CI)	P-value	Model 2 OR (95%CI)	P-value
OBS	0.97 (0.96,0.99)	0.003	0.98 (0.96,0.99)	0.008	0.98 (0.96,0.99)	0.006
OBS quartile						
Q1	ref	ref	ref	ref	ref	ref
Q2	0.74 (0.54,1.02)	0.064	0.76 (0.55,1.05)	0.092	0.77 (0.56,1.07)	0.116
Q3	0.65 (0.49,0.87)	0.004	0.68 (0.52,0.91)	0.009	0.67 (0.50,0.88)	0.005
Q4	0.65 (0.47,0.91)	0.013	0.69 (0.49,0.96)	0.027	0.69 (0.50,0.96)	0.027
P for trend		0.009		0.021		0.016
OBS. dietary	0.98 (0.96,1.00)	0.022	0.98 (0.96,1.00)	0.046	0.98 (0.96,1.00)	0.032
OBS. dietary quartile						
Q1	ref	ref	ref	ref	ref	ref
Q2	0.73 (0.52,1.02)	0.061	0.74 (0.53,1.04)	0.084	0.76 (0.54,1.06)	0.109
Q3	0.76 (0.56,1.02)	0.07	0.79 (0.59,1.07)	0.131	0.78 (0.58,1.04)	0.09
Q4	0.68 (0.49,0.96)	0.029	0.71 (0.51,1.00)	0.047	0.71 (0.52,0.99)	0.043
P for trend		0.048		0.083		0.06
OBS. lifestyle	0.82 (0.76,0.89)	<0.0001	0.84 (0.77,0.90)	<0.0001	0.85 (0.78,0.91)	<0.0001
OBS. lifestyle quartile						
Q1	ref	ref	ref	ref	ref	ref
Q2	0.62 (0.47,0.81)	<0.001	0.63 (0.48,0.84)	0.001	0.65 (0.49,0.85)	0.002
Q3	0.66 (0.51,0.86)	0.002	0.69 (0.53,0.90)	0.006	0.70 (0.54,0.91)	0.008
Q4	0.43 (0.30,0.62)	<0.0001	0.45 (0.31,0.66)	<0.0001	0.48 (0.33,0.70)	<0.001
P for trend		<0.0001		<0.0001		<0.001

The crude model did not adjust for any covariates; model 1 adjusted for age, sex, race/ethnicity, education, PIR, and marital status; and model 2 additionally adjusted for HbA1c, antidiabetic medication use, hypertension, CKD, TG, TC, and HDL-C from model 1. T2D, type 2 diabetes; OBS, oxidative balance score; CVD, cardiovascular disease; PIR, income-poverty ratio; HbA1c, glycated hemoglobin A1c; CKD, chronic kidney disease; TG, triglycerides; TC, total cholesterol; HDL-C, high-density lipoprotein cholesterol.

### 3.3 Association of OBS with all-cause and CVD mortality in the T2D population

**Figure 3:**
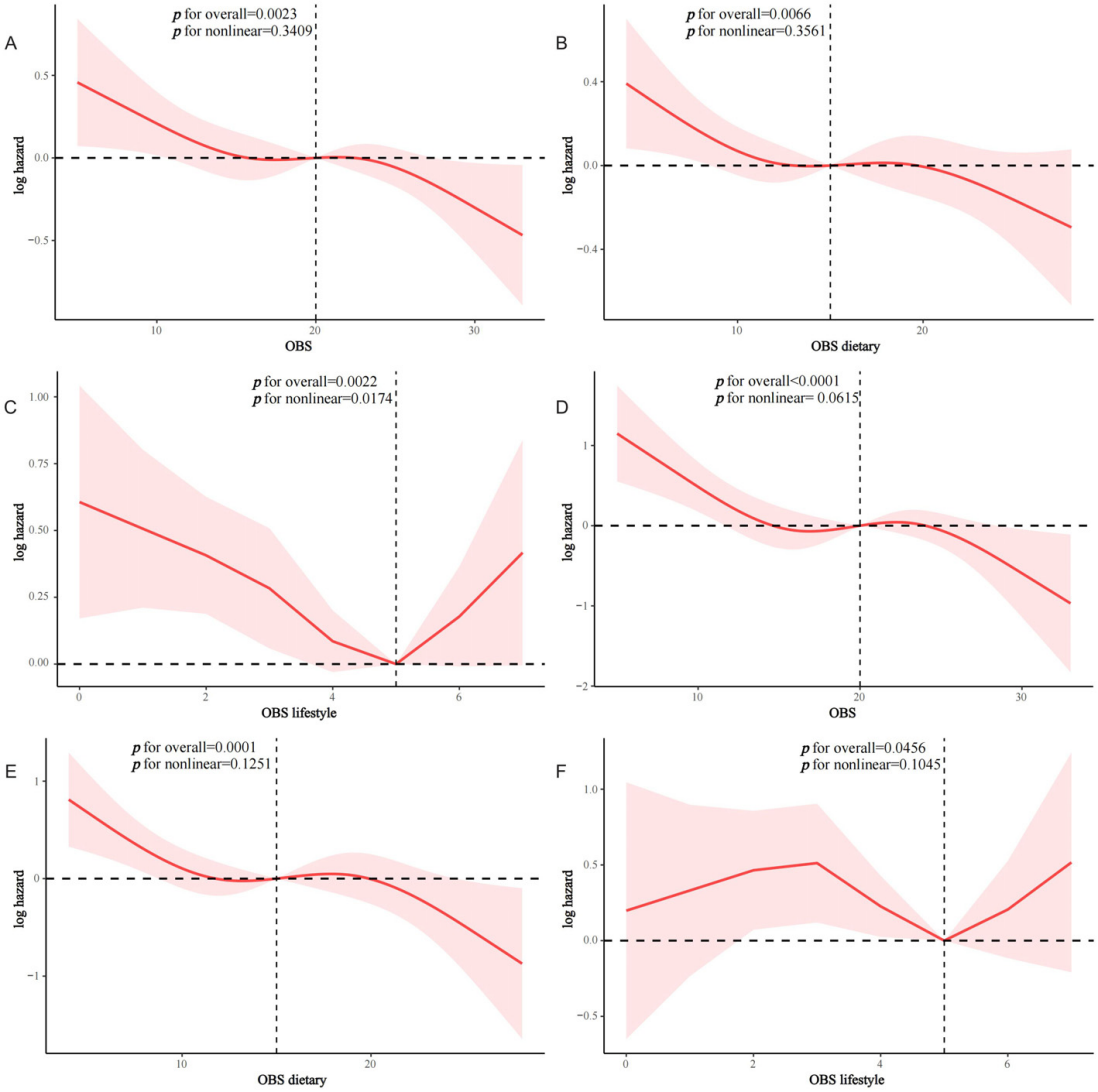
RCS analysis of OBS and mortality in the T2D population. **(A)** OBS and all-cause mortality; **(B)** dietary OBS and all-cause mortality; **(C)** lifestyle OBS and all-cause mortality; **(D)** OBS and CVD mortality; **(E)** dietary OBS and CVD mortality; **(F)** lifestyle OBS and CVD mortality.

After a median of 93 months of follow-up, 833 T2D patients died, of which 277 were CVD-related deaths (**Supplementary Table S2**). KM survival analyses showed significantly higher all-cause and CVD survival probabilities in the higher OBS quartiles compared to Q1 (both log-rank p < 0.0001) (**Supplementary Figures S1, S2**). In Model 2, OBS, dietary OBS, and lifestyle OBS were all negatively associated with all-cause mortality in the T2D population (OBS: hazard ratio [HR] and 95% CI = 0.98 (0.97,0.99), p < 0.001; dietary OBS: HR and 95% CI = 0.98 (0.97,0.99), p = 0.003; lifestyle OBS: HR and 95% CI=0.92(0.86,0.99), p=0.021). Higher OBS, dietary OBS, and lifestyle OBS were all associated with significantly lower all-cause mortality (p for trend 0.002, 0.009, and 0.035, respectively) (**Table 3**). In fully adjusted models, OBS and dietary OBS remained negatively associated with CVD mortality in the T2D population (OBS: HR and 95% CI = 0.96 (0.93,0.98), p < 0.001; dietary OBS: HR and 95% CI = 0.95 (0.93,0.98), p < 0.001), while lifestyle OBS lost the associations. Higher OBS and dietary OBS were associated with significantly lower CVD mortality (both p for trend <0.001) but not lifestyle OBS (p for trend = 0.085) (**Table 4**). RCS analysis suggested that most associations were linear, except for lifestyle OBS and all-cause mortality (p for nonlinearity = 0.0174) (**Figure 3**).

**Table 3:** Association of OBS with all-cause mortality in the T2D population.

	Crude Model HR (95%CI)	P-value	Model 1 HR (95%CI)	P-value	Model 2 HR (95%CI)	P-value
OBS	0.97 (0.96,0.98)	<0.0001	0.97 (0.96,0.99)	<0.0001	0.98 (0.97,0.99)	<0.001
OBS quartile						
Q1	ref	ref	ref	ref	ref	ref
Q2	0.82 (0.65,1.04)	0.101	0.84 (0.65,1.07)	0.152	0.85 (0.66,1.09)	0.202
Q3	0.78 (0.62,0.99)	0.04	0.81 (0.63,1.03)	0.084	0.88 (0.69,1.12)	0.291
Q4	0.57 (0.45,0.73)	<0.0001	0.60 (0.47,0.78)	<0.0001	0.67 (0.53,0.84)	<0.001
P for trend		<0.0001		<0.001		0.002
OBS. dietary	0.97 (0.96,0.98)	<0.0001	0.97 (0.96,0.99)	<0.001	0.98 (0.97,0.99)	0.003
OBS. dietary quartile						
Q1	ref	ref	ref	ref	ref	ref
Q2	0.78 (0.60,1.00)	0.048	0.79 (0.61,1.03)	0.082	0.86 (0.66,1.11)	0.238
Q3	0.74 (0.58,0.94)	0.013	0.76 (0.59,0.98)	0.037	0.84 (0.66,1.08)	0.179
Q4	0.60 (0.46,0.76)	<0.0001	0.62 (0.48,0.81)	<0.001	0.70 (0.55,0.90)	0.005
P for trend		<0.0001		<0.001		0.009
OBS. lifestyle	0.90 (0.85,0.97)	0.002	0.92 (0.86,0.98)	0.01	0.92 (0.86,0.99)	0.021
OBS. lifestyle quartile						
Q1	ref	ref	ref	ref	ref	ref
Q2	0.61 (0.48,0.77)	<0.0001	0.62 (0.48,0.79)	<0.001	0.64 (0.50,0.81)	<0.001
Q3	0.72 (0.56,0.94)	0.014	0.75 (0.57,0.97)	0.031	0.75 (0.58,0.98)	0.036
Q4	0.77 (0.59,1.00)	0.047	0.78 (0.61,1.02)	0.067	0.82 (0.63,1.06)	0.131
P for trend		0.008		0.017		0.035

The crude model did not adjust for any covariates; model 1 adjusted for age, sex, race/ethnicity, PIR, education, and marital status; and model 2 additionally adjusted for HbA1c, antidiabetic medication use, CVD, hypertension, CKD, TG, TC, and HDL-C based on model 1. T2D, type 2 diabetes; OBS, oxidative balance score; CVD, cardiovascular disease; PIR, income-poverty ratio; HbA1c, glycated hemoglobin A1c; CKD, chronic kidney disease; TG, triglycerides; TC, total cholesterol; HDL-C, high-density lipoprotein cholesterol.

**Table 4:** Association of OBS with CVD mortality in the T2D population.

	Crude Model HR (95%CI)	P-value	Model 1 HR (95%CI)	P-value	Model 2 HR (95%CI)	P-value
OBS	0.95 (0.93,0.97)	<0.0001	0.95 (0.93,0.98)	<0.0001	0.96 (0.93,0.98)	<0.001
OBS quartile						
Q1	ref	ref	ref	ref	ref	ref
Q2	0.61 (0.44,0.85)	0.003	0.62 (0.44,0.87)	0.006	0.60 (0.43,0.85)	0.004
Q3	0.68 (0.46,1.00)	0.053	0.68 (0.46,1.03)	0.065	0.74 (0.50,1.09)	0.123
Q4	0.35 (0.22,0.56)	<0.0001	0.36 (0.22,0.59)	<0.0001	0.39 (0.25,0.63)	<0.0001
P for trend		<0.0001		<0.001		<0.001
OBS. dietary	0.95 (0.93,0.97)	<0.0001	0.95 (0.92,0.98)	<0.001	0.95 (0.93,0.98)	<0.001
OBS. dietary quartile						
Q1	ref	ref	ref	ref	ref	ref
Q2	0.76 (0.51,1.14)	0.19	0.77 (0.50,1.20)	0.248	0.83 (0.54,1.27)	0.385
Q3	0.63 (0.42,0.93)	0.021	0.63 (0.40,0.98)	0.038	0.69 (0.45,1.06)	0.091
Q4	0.40 (0.24,0.66)	<0.001	0.41 (0.24,0.69)	<0.001	0.45 (0.27,0.73)	0.001
P for trend		<0.001		<0.001		<0.001
OBS. lifestyle	0.90 (0.81,1.00)	0.049	0.91 (0.82,1.02)	0.116	0.93 (0.83,1.03)	0.172
OBS. lifestyle quartile						
Q1	ref	ref	ref	ref	ref	ref
Q2	0.61 (0.44,0.84)	0.002	0.62 (0.45,0.86)	0.005	0.63 (0.45,0.89)	0.008
Q3	0.62 (0.41,0.94)	0.024	0.65 (0.42,1.00)	0.048	0.65 (0.43,0.99)	0.044
Q4	0.73 (0.47,1.14)	0.164	0.74 (0.48,1.15)	0.184	0.79 (0.52,1.21)	0.28
P for trend		0.039		0.062		0.085

The crude model did not adjust for any covariates; model 1 adjusted for age, sex, race/ethnicity, PIR, education, and marital status; and model 2 additionally adjusted for HbA1c, antidiabetic medication use, CVD, hypertension, CKD, TG, TC, and HDL-C based on model 1. T2D, type 2 diabetes; OBS, oxidative balance score; CVD, cardiovascular disease; PIR, income-poverty ratio; HbA1c, glycated hemoglobin A1c; CKD, chronic kidney disease; TG, triglycerides; TC, total cholesterol; HDL-C, high-density lipoprotein cholesterol.

### 3.4 Stratified analysis

**Figure 4:**
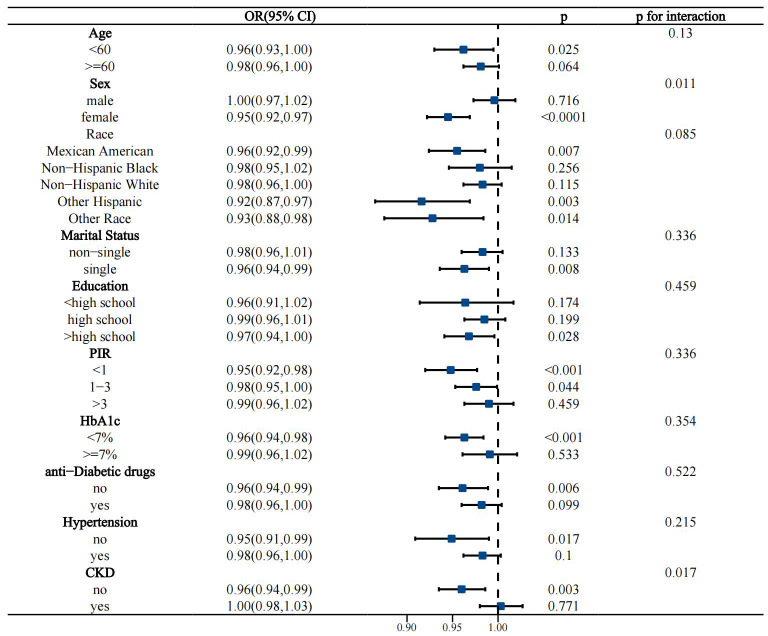
Stratified analysis of the association between OBS and the prevalence of CVD in the T2D population.

**Figure 5:**
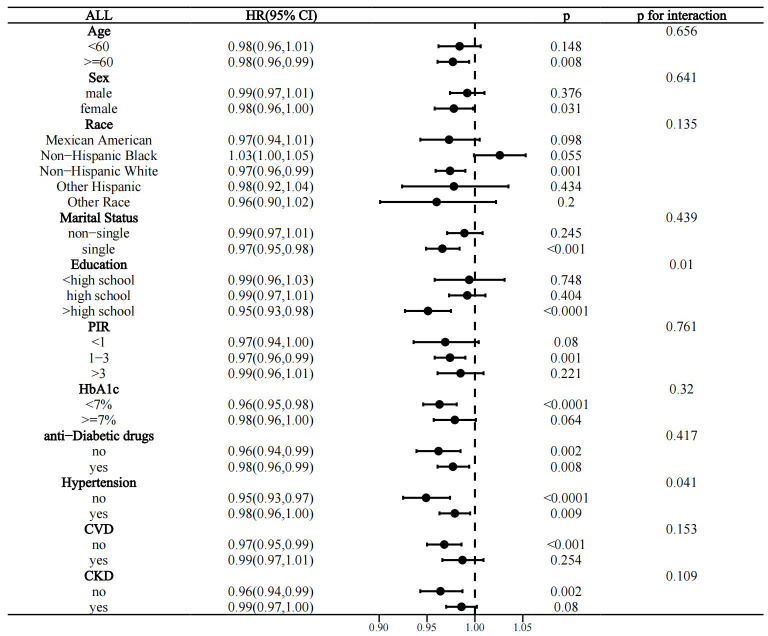
Stratified analysis of the association between OBS and all-cause mortality in the T2D population.

**Figure 6:**
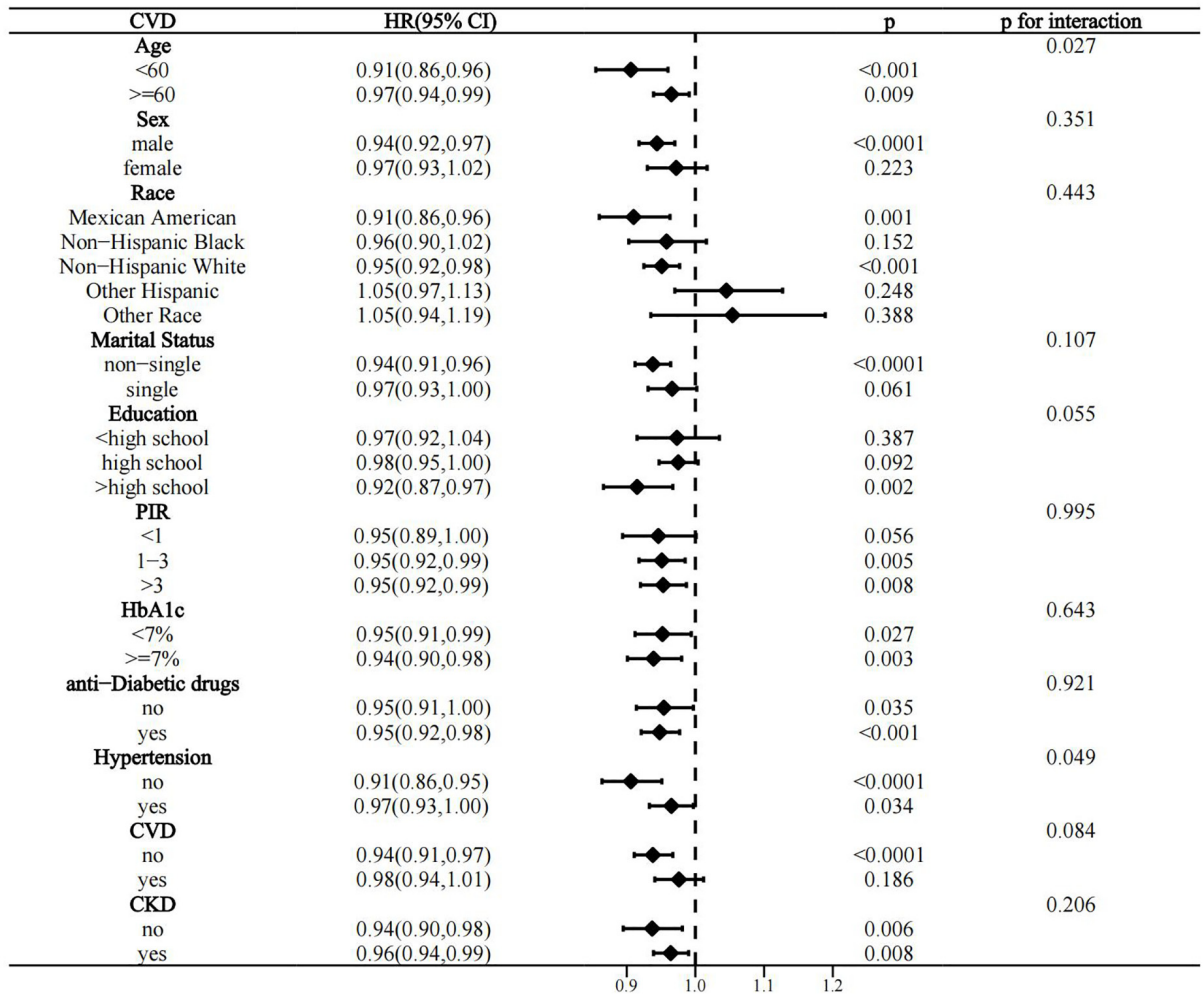
Stratified analysis of the association between OBS and CVD mortality in the T2D population.

Interactivity analysis showed that these associations were influenced by several factors. Gender and CKD were identified as effect modifiers of the association of OBS with CVD prevalence in the T2D population (p for interaction 0.011 and 0.017, respectively) (**Figure 4**). Educational level and hypertension significantly influenced the association between OBS and all-cause mortality (p for interaction 0.01 and 0.041, respectively) (**Figure 5**). Age and hypertension significantly influenced the association of OBS with CVD mortality (p for interaction 0.027 and 0.049, respectively) (**Figure 6**).

### 3.5 Sensitivity analysis

Additional adjustment for diabetes duration based on model 2 did not significantly change the results. OBS and lifestyle OBS remained associated with CVD prevalence in the T2D population (OR 0.98 and 0.83, respectively), although dietary OBS lost association (**Supplementary Table S3**). Similarly, OBS, dietary OBS, and lifestyle OBS remained significantly associated with all-cause mortality (HRs of 0.97, 0.98, and 0.87, respectively.) OBS and dietary OBS remained associated with CVD mortality, whereas lifestyle OBS remained unrelated (p=0.062) (**Supplementary Table S4**).

## 4 Discussions

In a national population-based study, we found that OBS was negatively associated with CVD prevalence, all-cause mortality, and CVD mortality in people with T2D. Specifically, dietary OBS was nonlinearly associated with CVD prevalence, whereas there was a dose-response association for lifestyle OBS. Dietary OBS was negatively and linearly associated with all-cause/CVD mortality, whereas lifestyle OBS had a significantly weaker association with mortality. These associations were influenced by several demographic aspects and disease conditions. Overall, these findings suggest that higher OBS is associated with reduced CVD prevalence and mortality risk in T2D populations. Interestingly, the beneficial effects of dietary and lifestyle OBS were each focused. Adherence to an antioxidant lifestyle was more significantly associated with CVD prevalence, whereas an antioxidant diet was more helpful in preventing all-cause and CVD mortality in the T2D population.

In this study, we used the OBS scheme that has been extensively validated in previous studies similarly using NHANES, including 16 dietary components and 4 lifestyle components, ensuring fit and consistency with the NHANES database (24, 25). Several clinical studies have explored the association between OBS and the occurrence of T2D in the general population. In a cross-sectional analysis, Wu et al. used NHANES 2007-2020 to show that OBS was negatively associated with the odds of T2D in the general U.S. adult population (fully adjusted OR = 0.96), with gender specificity (23). Kwon et al. demonstrated in a prospective cohort study that OBS was negatively associated with the incidence of T2D in the middle-aged and elderly Korean population, and that being in the highest OBS tertile was associated with a 17% and 22% lower risk of T2D in men and women, respectively (38). A cross-sectional analysis from Iran, on the other hand, showed that OBS was associated with better glycemic control in the T2D population (39). However, there are still a lack of research exploring the clinical prognostic relevance of OBS in people with diabetes. Our study demonstrated for the first time that OBS was negatively associated with CVD prevalence and mortality in the T2D population, providing new epidemiologic evidence for the public health significance of OBS in the T2D population.

Sparse observational clinical evidence suggests that OBS may be associated with CVD risk in general or specific populations, and there are inconsistent findings. A cross-sectional analysis using NHANES 2005-2018 suggested that OBS was negatively associated with the odds of CVD in the general US population (quartile Q4 compared to Q1: OR=0.67) and interacted with sleep patterns (16). A recent cross-sectional analysis similarly using NHANES 1999-2018 demonstrated that OBS was negatively associated with the odds of CVD in patients with NAFLD (OR=0.97, p=0.0015) (19). However, after adjusting for confounders, a previous cohort study did not confirm the association of OBS (including 12 dietary and lifestyle components) with CVD incidence (20). We speculated that these inconsistent findings may be due in part to differences in study populations, study designs, and OBS components. An interesting finding was that lifestyle OBS had a more robust association with the prevalence of CVD in T2D, which was in line with findings from several previous studies. A cross-sectional analysis from NHANES 2007-2018 indicated an interaction between systemic inflammatory markers in the association of OBS with specific types of CVD, whereas the association of lifestyle OBS with CVD in the general population was more pronounced (17). Similar findings were found in the association of OBS with the prevalence of CVD in the NAFLD population (19). In addition, we found that gender and CKD were significant effect modifiers, and this association was only observed in the female and CKD-free populations. A previous study similarly showed that gender influenced the association of OBS with the odds of T2D in the general population and was similarly more significant in women (compared to Q1, the OR for OBS at Q4 was 0.614 and 0.120 for men and women, respectively) (23). This may be partly explained by the differential role of sex-specific sex hormones on oxidative stress in the organism (23). Accumulating evidence suggests that the lower incidence of CVD among reproductive-age women is due in part to the cardiovascular beneficial effects of estrogen, and that estrogen and the estrogen receptor may exert CVD protection through mechanisms including oxidative stress (40). Thus, the unique estrogenic CVD-preventive effect in women may jointly with OBS reduce the odds of CVD in the T2D population. Recent studies have shown a negative correlation between OBS and CKD, thus CKD co-morbidities may lead to impaired oxidative homeostasis and diminish the effects of OBS (41). Compared to the CKD-free population, the CKD comorbid population has higher levels of oxidative stress, which results in cellular and organ damage that mediates the disease progression of CKD, including an increased incidence of CVD (42). Thus, the lower OBS levels and higher incidence of CVD in T2D patients with CKD may have contributed to the loss of the protective effect of OBS.

Our study demonstrated that OBS, particularly dietary OBS, was linearly and negatively associated with all-cause and CVD mortality in the T2D population, whereas the association of lifestyle OBS with mortality was less pronounced. Accumulating clinical studies have shown that OBS is negatively associated with mortality in the general population or in specific populations. A prospective cohort study from Spain demonstrated that OBS in the highest quartile (compared to the lowest quartile) was associated with significant reductions in all-cause (HR=0.35), CVD (HR=0.18), and cancer-related mortality (HR=0.35) in the general population (21). Another prospective cohort study from the US similarly showed that OBS was negatively associated with all-cause, cancer, and non-cancer mortality in the general population (43). Evidence from NHANES 2007-2018 similarly suggested that higher OBS was associated with lower all-cause mortality in the general U.S. older population (44). Other studies have suggested that OBS may be negatively associated with mortality in people with other metabolic diseases, such as NAFLD, metabolic syndrome, and dyslipidemia (19, 22, 45). Our study provides the first population-level evidence for OBS in the prevention of mortality in people with T2D. Our findings suggest that dietary OBS and overall OBS have a similar pattern of association with mortality in T2D, whereas the association was significantly weaker for lifestyle OBS. A previous cohort study showed a more significant association between lifestyle OBS and mortality in older women (46). This may indicate that the T2D population has distinctive clinical profiles compared to the general population that influence the relative impact of diet and lifestyle on mortality. In addition, the associations of OBS with all-cause and CVD mortality were more significant in those with > high school education level and in the nonelderly population, respectively. A previous study similarly demonstrated that the association of OBS with all-cause mortality in the NAFLD population was more significant in the highly educated population, suggesting that high educational level as an important socioeconomic status variable enhances the preventive value of OBS (19). Evidence from population-based studies suggests that higher socioeconomic status, including better education, is associated with lower all-cause and CVD mortality, possibly by influencing individuals’ access to knowledge resources, healthy lifestyles, and health care services (47). Older and younger adults with T2D have significantly different clinical characteristics (48), and we speculated that the younger population may have a higher long-term CVD mortality rate and therefore OBS may have more pronounced beneficial value. In addition, awareness of and adherence to healthy diets and lifestyles may be higher in younger age groups than in older age groups, which may partly explain this finding. In addition, the association of OBS with all-cause and CVD mortality in the T2D population was present only in people without hypertension, suggesting that the presence of hypertension may diminish the preventive value of OBS. People with hypertension exhibited significantly lower OBS than control populations and therefore had poorer antioxidant diet/lifestyle adherence (49). In addition, hypertension populations may have higher all-cause and CVD mortality rates (50), indirectly compromising the protective effect of OBS. Together, these findings suggest that adherence to OBS for the prevention of CVD and mortality in T2D populations requires individualized prevention strategies.

Recognizing that diabetes duration and antidiabetic medication use may influence these associations as important confounders, we considered these variables in fully adjusted models. After additional adjustment for diabetes duration, we found that OBS and lifestyle OBS remained negatively associated with the prevalence of CVD in the T2D population, whereas dietary OBS lost its association; furthermore, additional adjustment for diabetes duration did not significantly change the association of all OBS with mortality in the T2D population. Longer duration of diabetes has been shown to be associated with an increased risk of CVD in the diabetic population (51). Our findings suggest that diabetes duration may partially explain the protective effect of dietary OBS on the prevalence of CVD in the T2D population, i.e., dietary OBS may reduce the risk of CVD by reducing diabetes duration. Antidiabetic medication use did not significantly alter these associations, although there was a trend for these associations to be more pronounced in people without antidiabetic medication use. Although there is no relevant literature to support these findings, we speculate that this may be due to the fact that antidiabetic medication use, and antioxidant diet/lifestyle have a synergistic protective effect on CVD and mortality in the T2D population (52).

Although the mechanism of how OBS affects CVD and mortality in T2D populations remains unclear, it is conceivable that OBS may affect intrinsic oxidative homeostasis and thereby prevent disease. Several lines of clinical evidence suggest that higher OBS is associated with lower levels of oxidative stress markers, such as serum γ-glutamyltransferase (53) and urinary and plasma F2-isoprostane (54, 55). Thus, oxidative stress, an important contributor to CVD and clinical prognosis in the T2D population (9, 56), may be improved by better OBS compliance, partially explaining these findings.

Our study has several strengths. We comprehensively explored for the first time the association of OBS with CVD and mortality in the T2D population, which has potential public health implications. The nature of a large-sample national population-based study makes these findings potentially generalizable. We fully accounted for the influence of confounders and reduced potential study bias. However, there are limitations to our study. Most OBS components were assessed based on participant self-report and may be affected by recall bias. Consistent with previous studies (26), we recognize that equal weighting of all OBS components may underestimate or overestimate the antioxidant/pro-oxidant potential of the components. We did not have access to disease severity and other clinical characteristics of the T2D population and therefore could not adjust for the effects of these factors. The nature of observational studies prevents us from drawing causal relationships and is subject to residual confounding. It should be noted that there may be the possibility of reverse causality in these associations, i.e., people with T2D who have poorer health may adopt better lifestyles. However, there is also clinical evidence that people with CVD have lower adherence to healthy lifestyles (57). Means of ruling out reverse causal associations consist primarily of designing prospective cohort studies or randomized controlled trials. We adjusted for potential confounders as much as possible; however, given that the exploration of the association of OBS with CVD prevalence in the T2D population was based on cross-sectional analyses (limitations of the NHANES database), we could not rule out the possibility of reverse causality. However, the association between OBS and mortality in the T2D population was explored through a prospective cohort study (mortality information was derived from prospective matching with the National Death Index database), so reverse causation was less plausible. Future high-quality intervention studies are needed to validate these findings and determine potential causality.

## 5 Conclusions

Our findings suggested that OBS was negatively associated with CVD prevalence and risk of all-cause and CVD mortality in the T2D population. Adherence with higher dietary and lifestyle OBS was broadly associated with significantly lower CVD prevalence and mortality. Dietary OBS had a more pronounced association with mortality in the T2D population, whereas only lifestyle OBS had a dose-response association with CVD prevalence. Given that these findings are based on observational studies, these results merely represent the existence of an association and need to be interpreted with caution. Future high-quality randomized controlled trials are needed to validate these strategies and explore the possibility of applying them in clinical practice.

## Data Availability

Publicly available datasets were analyzed in this study. This data can be found here: This study analyzed publicly available datasets and can be found at https://www.cdc.gov/nchs/nhanes/.
