# Supplementary material for: Association of oxidative balance score with cardiovascular disease and all-cause and cardiovascular mortality in American adults with type 2 diabetes: data from the National Health and Nutrition examination survey 1999-2018
Source: Front Endocrinol (Lausanne). 2024 Dec 16;15:1458039. doi: 10.3389/fendo.2024.1458039 (PMC11682987; doi:10.3389/fendo.2024.1458039)

**Table S1.** Components and scoring approach for OBS, NHANES 1999-2018.

|  |  | Male |  |  | Female |  |  |
| --- | --- | --- | --- | --- | --- | --- | --- |
|  | Property | 0 | 1 | 2 | 0 | 1 | 2 |
| **Dietary OBS components** |  |  |  |  |  |  |  |
| Dietary fiber (g/d) | A | <12.55 | 12.55-19.67 | >19.67 | <10.05 | 10.05-16.33 | >16.33 |
| Carotene (RE/d) | A | <98.78 | 98.78-305.85 | >305.85 | <98.06 | 98.06-323.92 | >323.92 |
| Riboflavin (mg/d) | A | <1.79 | 1.79-2.73 | >2.73 | <1.34 | 1.34-2.12 | >2.12 |
| Niacin (mg/d) | A | <20.64 | 20.64-29.75 | >29.75 | <14.52 | 14.52-21.85 | >21.85 |
| Vitamin B6 (mg/d) | A | <1.59 | 1.59-2.40 | >2.40 | <1.13 | 1.13-1.77 | >1.77 |
| Total folate (mcg/d) | A | <315.46 | 315.46-491.5 | >491.5 | <250.90 | 250.90-388.5 | >388.5 |
| Vitamin B12 (mcg/d) | A | <3.35 | 3.35-6.20 | >6.20 | <2.22 | 2.22-4.21 | >4.21 |
| Vitamin C (mg/d) | A | <47.42 | 47.42-117.45 | >117.45 | <38.01 | 38.01-98.40 | >98.40 |
| Vitamin E (ATE) (mg/d) | A | <5.82 | 5.82-9.41 | >9.41 | <4.53 | 4.53-7.52 | >7.52 |
| Calcium (mg/d) | A | <645.50 | 645.50-1072.50 | >1072.50 | <499.00 | 499.00-848.50 | >848.50 |
| Magnesium (mg/d) | A | <256.50 | 256.50-361.02 | >361.02 | <186.53 | 186.53-283.00 | >283.00 |
| Zinc (mg/d) | A | <9.74 | 9.74-15.10 | >15.10 | <6.73 | 6.73-10.74 | >10.74 |
| Copper (mg/d) | A | <1.12 | 1.12-1.57 | >1.57 | <0.85 | 0.85-1.28 | >1.28 |
| Selenium (mcg/d) | A | <94.90 | 94.90-141.75 | >141.75 | <67.75 | 67.75-99.5 | >99.5 |
| Total fat (g/d) | P | >107.42 | 69.83-107.42 | <69.83 | >75.73 | 50.99-75.73 | <50.94 |
| Iron (mg/d) | P | >19.17 | 12.87-19.17 | <12.87 | >14.32 | 9.65-14.32 | <9.65 |
| **Lifestyle OBS components** |  |  |  |  |  |  |  |
| Physical activity (MET-minute/week) | A | <415.68 | 415.68-978.00 | >978.00 | <264.13 | 264.13-753.87 | >753.87 |
| Alcohol (drinks/d) | P | >30g/day | 0-30g/day | None | >15g/day | 0-15g/day | None |
| Body mass index (kg/m2) | P | >29.17 | 26.54-29.17 | <26.54 | >28.93 | 24.74-28.93 | <24.74 |
| Cotinine (ng/mL) | P | >1.13 | 0.04-1.13 | <0.04 | >0.17 | 0.04-0.17 | <0.04 |

**Table S2**. Follow-up and mortality data for the T2D population, NHANES 1999-2018.

|  | **Median (Q1-Q3)** | **Weighted** |
| --- | --- | --- |
| **Time, month (median, IQR)** | 93.00 (49.00-144.00) | 93.00 (49.00-144.00) |
| **All-cause death** | **N (%)** | **N** |
| alive | 2968(83.64) | 9901152 |
| death | 833(16.36) | 1937337 |
| **CVD-cause death** |  |  |
| No | 3524(94.60) | 11198695 |
| Yes | 277(5.40) | 639794 |

**Table S3**. Association of OBS with CVD prevalence in the T2D population after additional adjustment for diabetes duration based on model 2.

|  | **Model 3**  **OR (95%CI)** | **P-value** |
| --- | --- | --- |
| **OBS** | 0.98(0.96,1.00) | 0.04 |
| **OBS quartile** |  |  |
| Q1 | ref | ref |
| Q2 | 0.76(0.53,1.09) | 0.137 |
| Q3 | 0.64(0.45,0.90) | 0.01 |
| Q4 | 0.68(0.46,1.01) | 0.056 |
| **P for trend** |  | 0.036 |
| **OBS. dietary** | 0.98(0.96,1.01) | 0.136 |
| **OBS. dietary quartile** |  |  |
| Q1 | ref | ref |
| Q2 | 0.74(0.50,1.09) | 0.124 |
| Q3 | 0.74(0.52,1.05) | 0.091 |
| Q4 | 0.74(0.49,1.13) | 0.162 |
| **P for trend** |  | 0.171 |
| **OBS. lifestyle** | 0.83(0.76,0.91) | <0.001 |
| **OBS. lifestyle quartile** |  |  |
| Q1 | ref | ref |
| Q2 | 0.64(0.46,0.89) | 0.008 |
| Q3 | 0.69(0.51,0.95) | 0.023 |
| Q4 | 0.50(0.33,0.77) | 0.002 |
| **P for trend** |  | 0.002 |

Adjusted for age, sex, race/ethnicity, PIR, education, and marital status, HbA1c, antidiabetic medication use, CVD, hypertension, CKD, TG, TC, and HDL-C, and diabetes duration.

**Table S4**. Association of OBS with mortality in the T2D population after additional adjustment for diabetes duration based on model 2.

|  | **All-Cause**  **Model 3**  **HR (95%CI)** | **P-value** | **CVD Model 3**  **HR (95%CI)** | **P-value** |
| --- | --- | --- | --- | --- |
| **OBS** | 0.97(0.96,0.99) | 0.003 | 0.94(0.92,0.97) | <0.001 |
| **OBS quartile** |  |  |  |  |
| Q1 | ref | ref | ref | ref |
| Q2 | 0.86(0.65,1.15) | 0.314 | 0.76(0.49,1.18) | 0.217 |
| Q3 | 0.89(0.67,1.17) | 0.393 | 0.72(0.45,1.18) | 0.19 |
| Q4 | 0.59(0.42,0.82) | 0.002 | 0.28(0.15,0.52) | <0.0001 |
| **P for trend** |  | 0.007 |  | <0.001 |
| **OBS. dietary** | 0.98(0.96,1.00) | 0.016 | 0.94(0.91,0.97) | <0.001 |
| **OBS. dietary quartile** |  |  |  |  |
| Q1 | ref | ref | ref | ref |
| Q2 | 0.81(0.60,1.11) | 0.186 | 0.89(0.54,1.44) | 0.628 |
| Q3 | 0.77(0.57,1.03) | 0.077 | 0.59(0.35,0.99) | 0.045 |
| Q4 | 0.68(0.48,0.96) | 0.026 | 0.40(0.21,0.78) | 0.007 |
| **P for trend** |  | 0.032 |  | 0.002 |
| **OBS. lifestyle** | 0.87(0.81,0.94) | <0.001 | 0.89(0.79,1.01) | 0.062 |
| **OBS. lifestyle quartile** |  |  |  |  |
| Q1 | ref | ref | ref | ref |
| Q2 | 0.56(0.42,0.75) | <0.0001 | 0.54(0.34,0.85) | 0.007 |
| Q3 | 0.61(0.44,0.84) | 0.003 | 0.63(0.38,1.04) | 0.073 |
| Q4 | 0.68(0.52,0.88) | 0.003 | 0.64(0.38,1.08) | 0.096 |
| **P for trend** |  | <0.001 |  | 0.05 |

Adjusted for age, sex, race/ethnicity, PIR, education, and marital status, HbA1c, antidiabetic medication use, CVD, hypertension, CKD, TG, TC, and HDL-C, and diabetes duration.

**Figure S1**. KM survival analysis of OBS and all-cause survival probabilities in the T2D population.


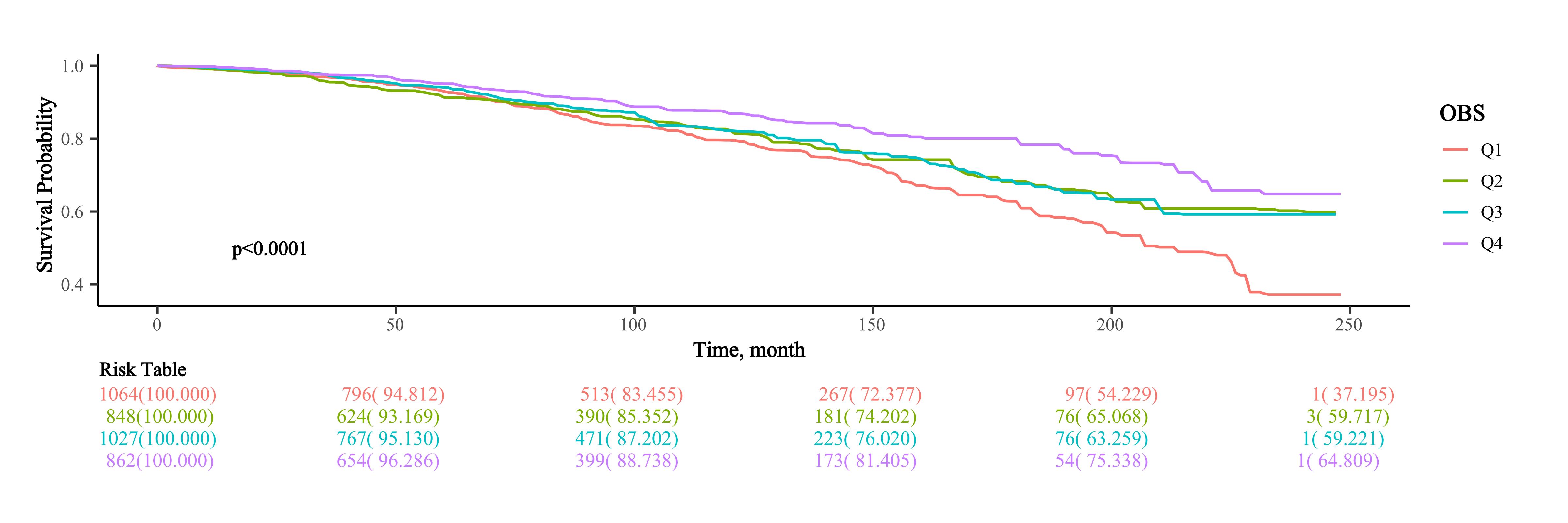


**Figure S2**. KM survival analysis of OBS and CVD survival probabilities in the T2D population.


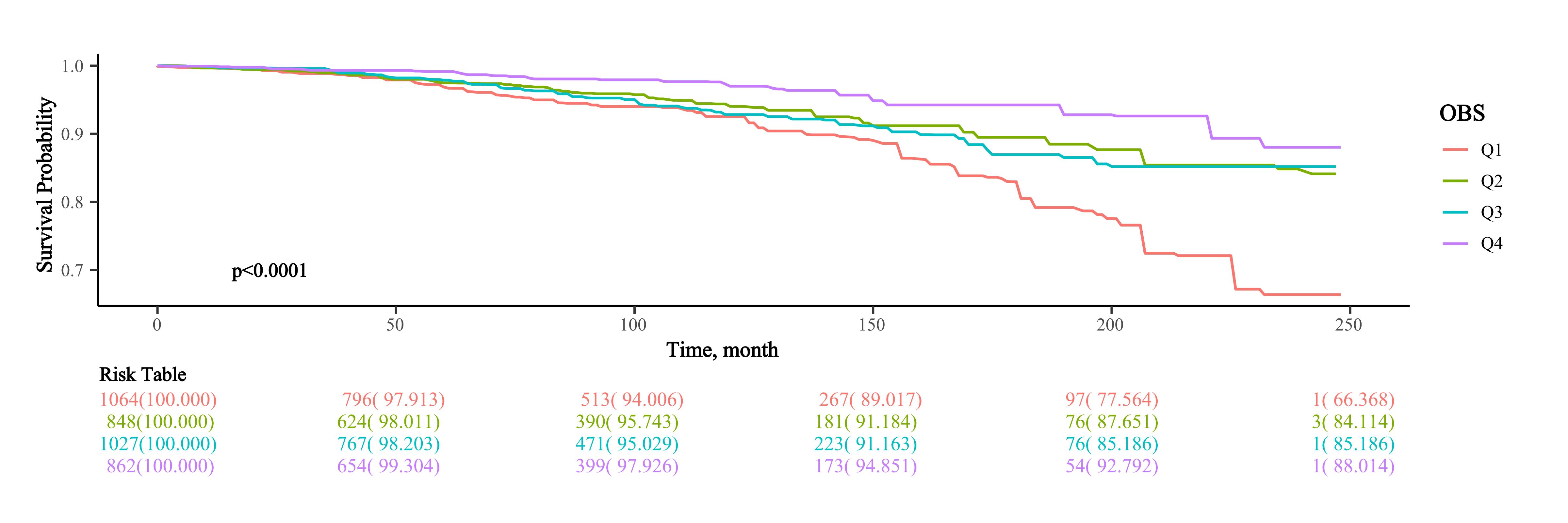

Supplement: Supplementary file 1 [file DataSheet1.docx]
